# Watching the Watchman

**Published:** 1892-03

**Authors:** 


### WATCHING THE WATCHMAN.

The N. Y. *Medical Record* says that in the drugstore, in New York, where Medical Student Carlyle Harris (recently convicted of uxoricide), had the fatal morphine capsules put up, it so happened, accidentally or by design, the *Record* says not, that while the druggist was filling the prescription, another druggist clerk was looking over his shoulders; and at the trial testified to the correct filling of the prescription; otherwise Harris could have claimed that there had been a mistake made by the druggist, and thus, throwing the blame on the druggist, would have gone "unwhipped of justice" for his dastardly and infamous crime.

The *Record* suggests that druggists profit by the narrow escape of their New York brother, and establish a rule ln every dispensing drugstore, that it shall require two clerks to fill each prescription calling for a poisonous drug; one to fill and one to look on, over his shoulder, acting thus as a tally or check on the filler.

The druggist has always been regarded as the check on the hasty, incompetent, or thoughtless doctor, and numerous instances have occurred where he has detected an overdose ordered, or an incompatible mixture, and calling the doctor's attention to it, has doubtless prevented much killing. But, now, druggists are getting to be careless, or unreliable, and the watchman must himself be watched. Then, bye and bye, may be watchman No. 2 will get dazed or careless; and while ostensibly looking over No. 1's shoulder ("over the left"), he may be catching flies, or thinking of his sins, or falling into a fit of abstraction over last night's festivities, and "the both of 'em" may let slip a mistake; and a watchman will be required for him who was set to watch the watchman. Where, then, will this thing end?

Congress took a hand in the effort to prevent druggists dispensing morphine for quinine, and ordered all packages of morphine to be put up in *red*. It was thought for a while that this had stopped the trouble; for a druggist in the presence of his senses could hardly take up a blazing red package without knowing it. But all the same the mistakes go on, and get in their deadly work.

The JOURNAL knows a druggist who has a record of never having made a mistake in dispensing. He has a plan of his own, a check on himself. He takes the articles in the prescription *seriatim*, and checks them off, setting the container to the right. When the ingredients are weighed out, each article is checked back, before the container is restored to the shelf. This, at least, seems to concentrate the attention of the druggist on what he doing, and we opine, the mistakes alluded to are mostly made in consequence of inattention or abstraction ; but,—

WHY NOT abolish the practice altogether, of having another man dispense your medicine, as the JOURNAL insisted in last issue ? The danger of mistakes on the part of the clerk is alone sufficient justification; but there are many other reasons, as set forth in the article alluded to. And since it has become entirely unsafe to risk a single man to fill a prescription, and he needs must have an overseer to watch him, there can be no sound reason in opposition to our position, and in favor of continuing the antiquated, tedious, unreliable, expensive, and altogether farcical practice of prescribing medicines to be put up at the junk shop of the patent medicine vendor,—the counter-prescriber, the physician's secret enemy. Doctors, have either of you who read this, ever had a patient to call for you at a drugstore, to ask if he shall have your last prescription refilled, and that patient sent away, without seeing you, yet with the bottle re-filled, or with a bottle of something the druggist has sold him in lieu of it ? We repeat and strongly insist that it is to the best interest of the medical profession, and of the sick, that physicians shall dispense their own medicines at the bedside; put out enough (it can all be had now in concentrated form, and ready and convenient for administration), to last till next visit ; and there will be none of those vexatious delays, no accidents and no drug bill to pay to your patent medicine rival-for-practice, who is using every means in his power to undermine and break down the regular practice of medicine, and to build up on its ruins the sale of his own or some other secret nostrum.

---

THE Massachusetts Society for the Prevention of Cruelty to Animals has a standing reward of $100, for evidence to convict any person in that State of maiming a horse for life, by the barbarous practice of what is known as "docking."

Would that there were some society in Texas, for a similar purpose. In our opinion, clipping a horse in winter is just as bad, if not worse than docking ; it is the refinement of cruelty. Yet, at the capital, in the *Daily Statesman*, one may read of an establishment right here, where it is made a business, and it is announced that you can have your horse clipped (stripped of his protection against the norther), "while you wait."

